# Anticorrosive non-crystalline coating prepared by plasma electrolytic oxidation for ship low carbon steel pipes

**DOI:** 10.1038/s41598-020-72787-w

**Published:** 2020-09-24

**Authors:** Chunsheng Ma, Jian Liu, Xinhe Zhu, Wenbin Xue, Zhijun Yan, Dong Cheng, Jingguo Fu, Shenglin Ma

**Affiliations:** 1 College of Marine Engineering, Dalian Maritime University, Dalian, 116026 China; 2 College of Nuclear Science and Technology, Beijing Normal University, Beijing, 100875 China

**Keywords:** Design, synthesis and processing, Surfaces, interfaces and thin films

## Abstract

A corrosion-resistant non-crystalline coating was fabricated by plasma electrolytic oxidation (PEO) on Q235 low carbon steel for ship pipes. The distribution and composition of chemical elements and phases of PEO coatings were analyzed by an orthogonal experiment, and the formation mechanism of PEO coatings was discussed. The corrosion current densities and corrosion potentials were measured. The results indicated that the formation of a transition layer mainly containing Fe_3_O_4_ was crucial for achieving an excellent coating quality. Furthermore, the corrosion current density of coated steel was reduced by 78% compared with the bare steel.

## Introduction

Low carbon steel (LCS) is one of the most widely employed metal materials in the shipping industry due to good plasticity and ductility, and low cost^1,2^. For instance, the majority of ship pipeline is fabricated by LCS^3^. However, LCS has weak corrosion resistance^4^. To improve reliability and durability of ship LCS pipeline, a large number of technologies have been employed for LCS pipes, such as electrogalvanizing, hot-dip galvanizing, powder zinc impregnation, rubber-coated pipes, and fiberglass pipes^5–8^. Nevertheless, the existing technologies have various disadvantages, for instance, environmental pollution and health problems caused by electrogalvanizing and hot-dip galvanizing, the powder zinc impregnation is not suitable for pipes due to the requirement of strict surface pretreatments, and high cost introduced by rubber-coated and fiberglass pipes.

The plasma electrolytic oxidation (PEO) is one of the most promising techniques to fabricate anticorrosive coating for the LCS pipeline^1^. The PEO is widely used to fabricate functional coatings on light metals and their alloys, such as Al, Mg, and Ti^9–16^. During PEO processes, the substrate of samples does not endure a high thermal load. Because the arc discharge only happens in localized zones and remains for a very short period^17^. Meanwhile, the requirement of pretreatments for PEO is not strict due to the high energy of the plasma arc. Furthermore, the PEO can achieve metallic bonding between the substrate and coatings^18^. Therefore, the PEO is suitable to carry out inside surface anti-corrosion treatment for ship LCS pipes.

At present, there are three types of techniques to fabricate PEO coatings on the surface of carbon steel, including direct PEO, PEO on hot-dip aluminized carbon steel, and PEO after the deposition of Al_2_O_3_ or SiO_2_ layer on the surface of carbon steel^2,19–21^. Zhaohua Jiang et al. mainly achieved four kinds of PEO coatings in different electrolytes. The four types of ceramic coatings are Fe_3_O_4_, amorphous coating, Fe_3_O_4_ and FeAl_2_O_4_, and FeAl_2_O_4_, Fe_3_O_4_, and a little γ-Al_2_O_3_^22–27^. Jun Liang et al. prepared a ceramic coating composed of SiO_2_, Fe_2_O_3_, Fe_3_O_4_ in silicate electrolyte with the additive of Al nanoparticles, the corrosion resistance of the coating was improved significantly^1^. Malinovschi et al. obtained an amorphous SiO_2_ coating on the surface of S234JR steel in sodium silicate/carbonate electrolyte; the coating has a certain corrosion resistance^2^.

For ship LCS pipes, the ceramic coatings composed of iron oxide and aluminum oxide are not suitable for corrosion protection against acid environment. For example, it is necessary to have excellent acid corrosion resistance for the wastewater pipes of scrubbers on ships. As well known, SiO_2_ coating has excellent corrosion resistance against acid. However, the preparation of SiO_2_ ceramic coatings has not been studied thoroughly; the fabrication process and formation mechanism of SiO_2_ coatings are not clear. The barriers constraint the application of PEO technology in the surface treatment of low carbon steel.

In this work, an orthogonal experiment was employed to thoroughly analyze the preparation process and formation mechanism of the SiO_2_ PEO coating, which is significant for further promotion of the SiO_2_ coating. The relationship of main PEO electric parameters, chemical composition and compactness of the PEO coatings is firstly revealed. X-Ray Photoelectron Spectroscopy (XPS) mapping and Glow Discharge Optical Emission Spectrometer (GDOES) were performed for chemical composition analysis of PEO coatings. Finally, the corrosion resistance of PEO coatings was evaluated.

## Experimental details

The Q235 carbon steel samples (100 mm × 10 mm × 5 mm) were used to fabricate PEO coatings by a self-developing power supply with a duty cycle of 50%. The PEO electrolyte was composed of Na_2_SiO_3_·5H_2_O (Kermel, AR), Na_2_CO_3_ (15 g/L, Kermel, AR), and DI water. The cathode is a stainless steel tank (15 L). A cooling system is performed to maintain the electrolyte temperature within about 34 °C on a water chilling unit and some cooling water circulation lines.

An orthogonal experiment of three factors and four levels was designed. Because the positive voltage and frequency are the main electric parameters, and the Na_2_SiO_3_ is a typical electrolyte for PEO treatment, they were chosen to optimize the PEO process. As the PEO breakdown voltage (BV) varies with the changing of frequency and concentration of sodium silicate, the levels of positive voltage were set as BV + 40 V, BV + 50 V, BV + 60 V, and BV + 70 V.The breakdown voltages were recorded by the appearance of rapid drop in current. The levels of sodium silicate concentration are based on lots of previous experiments and the literature^2^. The protocol for the orthogonal experiment is shown in Table 1. The PEO reaction time was chosen as 5 min from the positive voltage reaching the levels of the Positive Voltage, as shown in Table 1.

**Table 1:** The protocol of the orthogonal experiment for fabricating PEO coatings.

Test code	Positive voltage (V)	Frequency (Hz)	Concentration of sodium silicate (g/L)
1	BV + 40	500	19
2	BV + 40	1000	21
3	BV + 40	1500	23
4	BV + 40	2000	25
5	BV + 50	500	21
6	BV + 50	1000	23
7	BV + 50	1500	25
8	BV + 50	2000	19
9	BV + 60	500	23
10	BV + 60	1000	25
11	BV + 60	1500	19
12	BV + 60	2000	21
13	BV + 70	500	25
14	BV + 70	1000	19
15	BV + 70	1500	21
16	BV + 70	2000	23

To achieve the deposition of SiO_2_ before the PEO process, in the beginning, the positive voltage was increased from 50 to 100 V in 5 V increments every 1 min. Then, it was fast raised to the levels of Positive Voltage shown in Table 1. This operation is based on the literature^2^.

Surface analysis of PEO coatings performed by X-Ray Photoelectron Spectroscopy (XPS) on phi5000VersaProbe. The thickness, surface, and cross-section morphologies of PEO coatings were detected by a scanning electron microscope (SEM, VEGA 3, TESCAN). X-ray diffraction (XRD, EMPYREAN) was employed to analyze the phase composition of PEO coatings. X-ray diffraction patterns were collected using CoK_α_ radiation: the accelerating voltage is 35 kV, scan range 2θ is 15°–130°, and step size Δ2θ is 0.039°. To analyze the patterns, the X-ray diffraction patterns were transformed to Cu-target types finally. The surface roughness and three-dimensional shape of the coatings were analyzed by a 3D measuring laser microscope (OLYMPUS, OLS4000). The element composition and distribution across coatings were detected by a glow discharge optical emission spectrometer (GDOES, SPECTRUMA, GDA 750HR) with a 2.5 mm anode. The electrochemical corrosion was evaluated by potentiodynamic polarization measurements. The polarization curves were obtained by an electrochemical system (Shanghai Chenhua, CHI604E) with 3.5 wt% NaCl and a scanning rate of 0.002 V/S. Five samples of each set of conditions were tested for each data, so as to avoid the fluctuations in the data, and the reported values are the average resulted from these measurements.

## Results and discussion

### The coating-forming process

As shown in Table 2, the changes of thickness and surface roughness of PEO coatings are not significant in the orthogonal experiment. According to the range analysis method, the sequence of the three factors for the thickness of coatings is the positive voltage (range 9.37), the concentration of sodium silicate (range 5.15), and the frequency (range 2.76). The sequence for the surface roughness is also the positive voltage (range 0.44), the concentration of sodium silicate (range 0.14), and the frequency (range 0.13). Therefore, the positive voltage is the most significant factor for the preparation of PEO coatings. Simultaneously, the thickness and surface roughness become higher with increasing the positive voltage due to the high energy of plasma arcs. Hence, the positive currents in the last 5 min of PEO processes are higher while the difference between the positive voltage and breakdown voltage are more significant, as shown in Table 3.

**Table 2:** The thickness and surface roughness of PEO coatings.

Test code	Thickness (μm)	Surface roughness (Ra, μm)
1	7.88	2.80
2	13.14	2.75
3	15.90	2.68
4	17.75	2.67
5	19.27	3.08
6	19.07	3.11
7	23.07	2.93
8	16.04	2.99
9	20.97	3.25
10	19.02	3.00
11	19.25	3.01
12	22.40	3.11
13	23.54	3.09
14	19.63	3.28
15	23.25	3.31
16	25.75	2.95

**Table. 3:** The positive voltage and range of positive current in the last 5 min of PEO processes.

Test code	Positive voltage (V)	Range of positive current (A)
1	BV + 40 = 180	35.2 ~ 9.2
2	BV + 40 = 180	32.8 ~ 8.6
3	BV + 40 = 180	37.6 ~ 11.5
4	BV + 40 = 160	30.6 ~ 14.9
5	BV + 50 = 190	43.3 ~ 9.3
6	BV + 50 = 190	42.6 ~ 7.1
7	BV + 50 = 160	40.6 ~ 9.4
8	BV + 50 = 220	43.3 ~ 8.5
9	BV + 60 = 170	42.4 ~ 9.2
10	BV + 60 = 170	47.7 ~ 10.1
11	BV + 60 = 200	43.8 ~ 14.5
12	BV + 60 = 190	44.2 ~ 11.6
13	BV + 70 = 190	46.7 ~ 8.0
14	BV + 70 = 190	44.6 ~ 12.2
15	BV + 70 = 190	46.4 ~ 14.5
16	BV + 70 = 190	45.6 ~ 10.1

**Figure 1:**
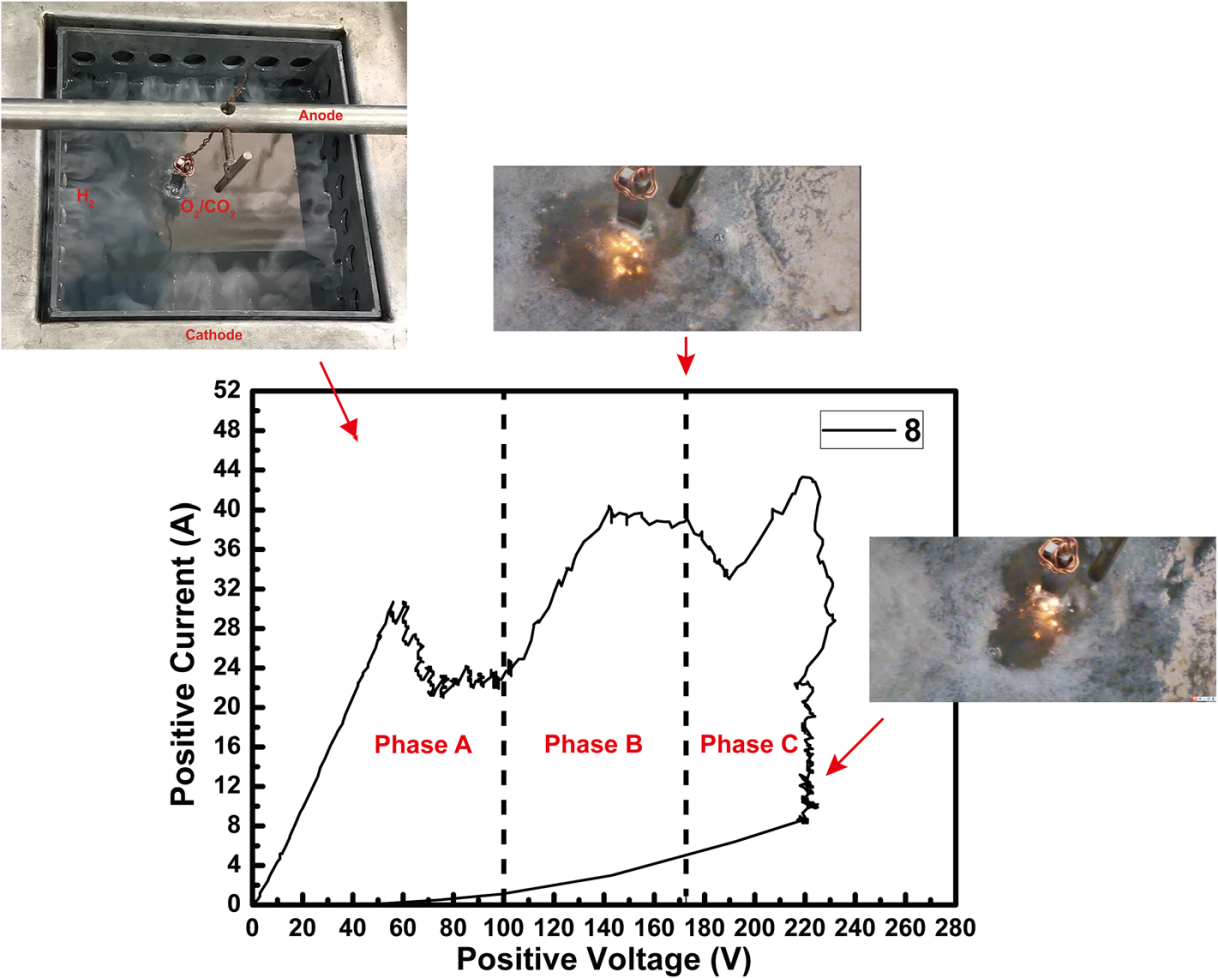
The current–voltage diagram for the PEO process of Test 8.

The changes of positive current with increasing the positive voltage for Test 8 are shown in Fig. 1.

The PEO process can be divided into three phases: Phase A, Phase B, and Phase C. In Phase A, the positive voltage is increased from 50 to 100 V with 5 V increments per 1 min; during the Phase A, the anode produces a mass of oxygen accompanied with emitting a large number of bubbles; meanwhile, the cathode produces a large amount of hydrogen.

Meanwhile, a SiO_2_ film is also deposited on the surface of the anode, according to the literature^2^. The SiO_2_ and oxygen film are crucial for the PEO reaction, as they provide discharge channels for the PEO process. Because the SiO_2_ passive film is produced on the anode, the current decreases from 30 A to around 22 A in Phase A. After Phase A, the positive voltage increases manually very fast. Until about 170 V, the anode surface exhibits an evident discharge phenomenon, as shown in Phase B. Therefore, the 170 V is the breakdown voltage for Test 8. Then the positive voltage is manually raised to 220 V very fast in Phase C, according to the protocol of the orthogonal experiment. The primary PEO process starts from the positive voltage reaching 220 V for Test 8. During the 5 min, the positive current presented in Table 3 decreases from 43.3 A to 8.5 A. By the end of the PEO process, the discharge becomes more and more intense due to the coating growth, the microarcs become more large and bright, but the density of microarcs decreases correspondingly as shown in the Fig. 1. The other samples of the orthogonal experiment also exhibit a similar reaction phenomenon with Test 8 during PEO processes, but the values of positive current are different from each other (see in Table 3). Because the thickness and compactness of the SiO_2_ passive film affect the difficulty level of breakdown, they are also determined by the frequency and concentration of sodium silicate. Therefore, the PEO coatings exhibit kinds of characteristics in the orthogonal experiment, such as elements distribution, morphologies, and phase constituents.

**Figure 2:**
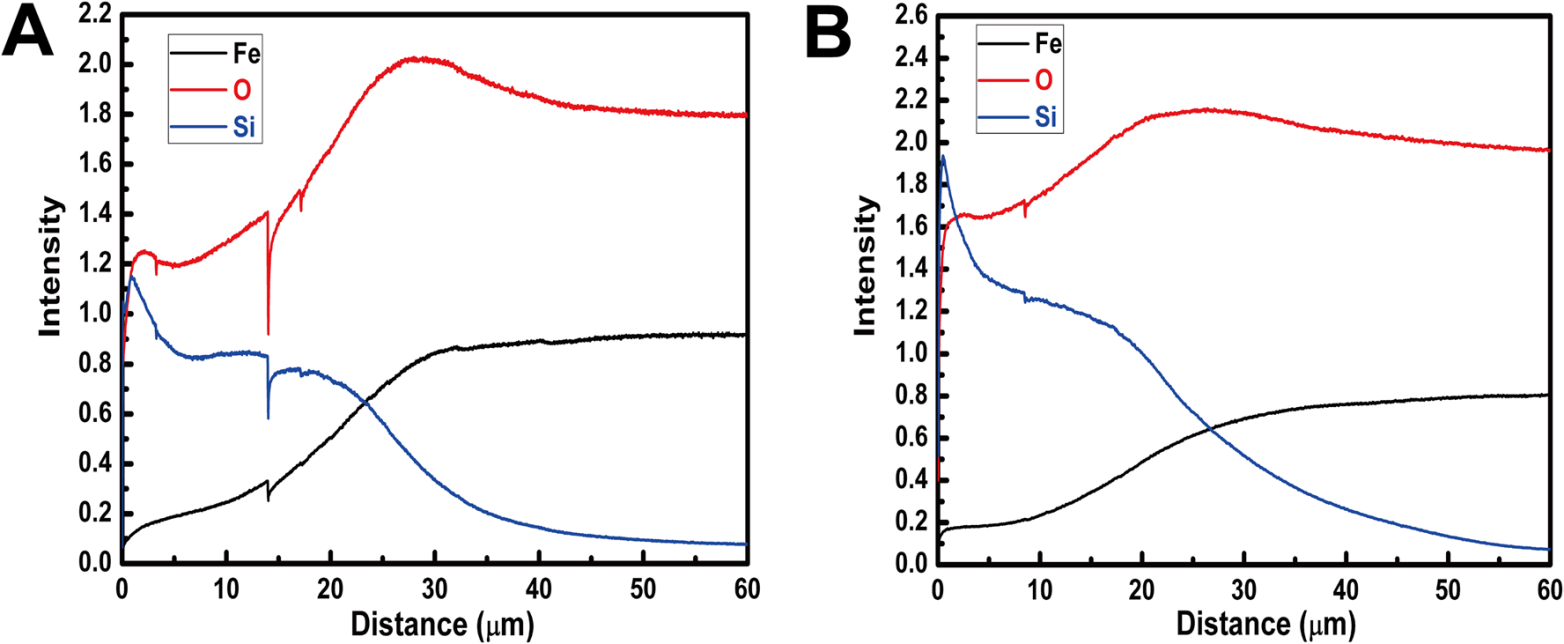
The elements distributions of cross-section areas: a Test 4, and B Test 16.

The composition profiles of elements for the PEO coatings of Test 4 and 16 are shown in Fig. 2.

Because the stand sample was not fabricated and detected, the values of Fig. 2 are only for analyzing the changing trends of each element. According to the results of GDOES (Glow Discharge Spectrometer), the main elements contained in the PEO coatings are Fe, O, and Si; the contents of other elements approach zero. Moreover, from the surface to the steel substrate of the samples, the elements (Fe, O, Si) are not evenly distributed, as shown in Fig. 2. The volatility of the elements content is from the current volatility of the equipment. The surface of PEO coatings mainly contains the elements of Si and O; meanwhile, the content of Si decreases, and the content of Fe increases from the surface to the steel substrate. Moreover, the O and Fe have similar changing trends, so the Fe and O work together in the PEO coatings. While the X-ray reaches the substrate of samples, the content of Fe, O, and Si are stable. Therefore, the steel substrate (Fe) also participates the PEO reaction. Moreover, the oxidation of Fe plays a dominant role near the bonding surface, and the role becomes weaker while approaching the surface of PEO coatings. On the other hand, the formation of SiO_2_ plays a primary role in the outer layer of the PEO coatings.

### The morphology and composition of PEO coatings

**Figure 3:**
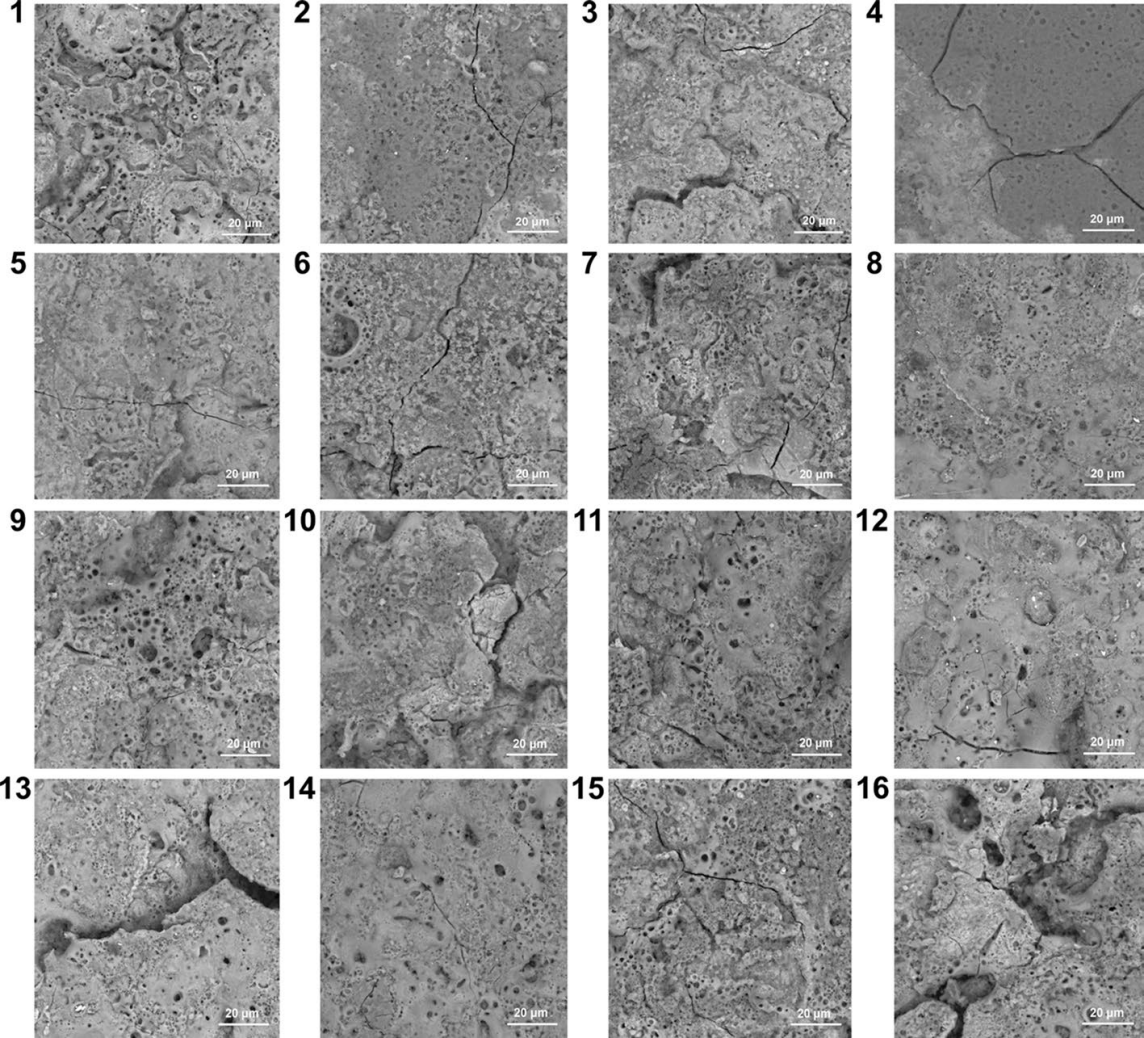
The PEO coatings surface morphologies of the orthogonal experiment.

**Figure 4:**
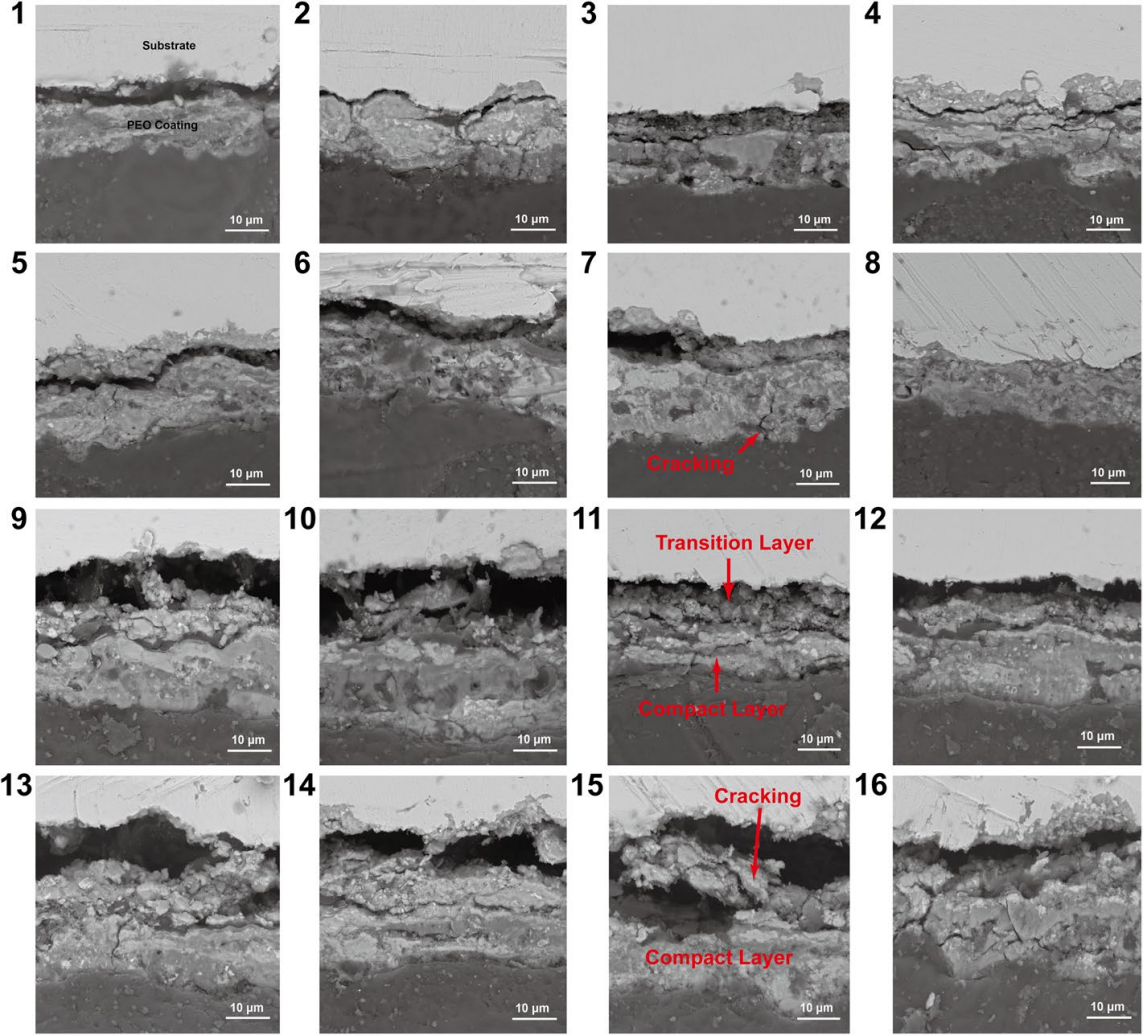
The PEO coatings cross-section morphologies of the orthogonal experiment.

The surface morphologies of PEO coatings are shown in Fig. 3. The cross-section morphologies of PEO coatings are given in Fig. 4.

As shown in Fig. 3, the PEO coatings exhibit similar surface morphologies with the traditional PEO coatings (Crater-shaped). However, most of PEO coatings have some defects, such as cracking and remained large discharge channels, which are not suitable for anti-corrosion. Meanwhile, as shown in Fig. 4, the majority of PEO coatings do not have excellent adhesion conditions. However, the PEO coating prepared in Test 8 has a smooth surface morphology and a good adhesion condition with the steel substrate. To analyze deeply, the XRD (X-ray Diffraction) is carried out for detecting the phase compositions of the PEO coatings.

**Figure 5:**
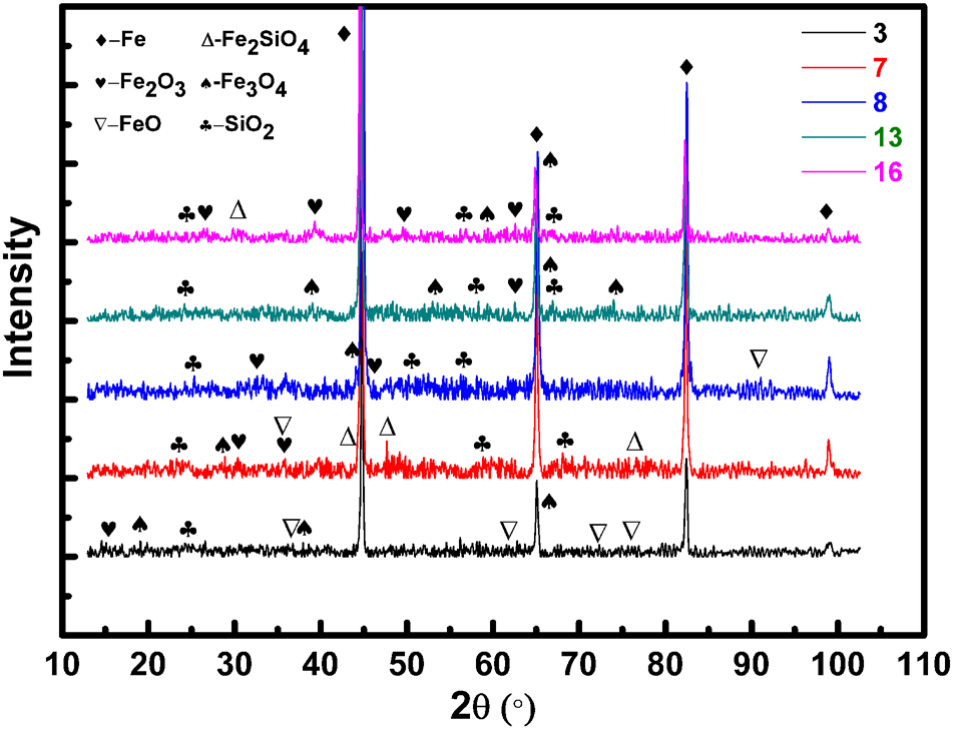
XRD patterns of PEO coatings prepared in: Test 3, 7, 8, 13, and 16.

Figure 5 exhibits the phase compositions of the PEO coatings of Test 3, 7, 8, 13, and 16.

The main peaks in Fig. 5 are for Fe. Because the PEO coatings are relatively thin, the X-ray already reaches the substrate of samples. Apart from the peaks for Fe, there are several weak peaks for Fe_2_O_3_, Fe_3_O_4_, FeO, Fe_2_SiO_4_, and SiO_2_. As the peaks have much lower intensity, these phases do not have high crystallinity, and the grains dimension are relatively small. Therefore, the PEO coatings are a kind of non-crystalline coatings. It is observed in Fig. 5 that there is a small bread peak at around 2θ = 25° in the XRD curve of sample 3 and 7. The bread peaks are ascribed to amorphous SiO_2_. However, bread peaks are not detected in the curves of sample 8, 13, and 16. Furthermore, there are several lower peaks of SiO_2_ in the XRD patterns of sample 8, 13, and 16. Therefore, the PEO coatings prepared in Test 8, 13, and 16 have higher crystallinity. Moreover, the XRD curve of sample 8 contains higher intensity of peaks than the other curves, for instance, at around 2θ = 65.2° there is a peak for Fe_3_O_4_, which has the highest intensity of Fe_3_O_4_ peaks. Thus, the PEO coating produced in Test 8 has the highest crystallinity among these coatings. This is one reason for the PEO coating produced by Test 8 exhibiting relatively excellent surface morphology and adhesion condition.

**Figure 6:**
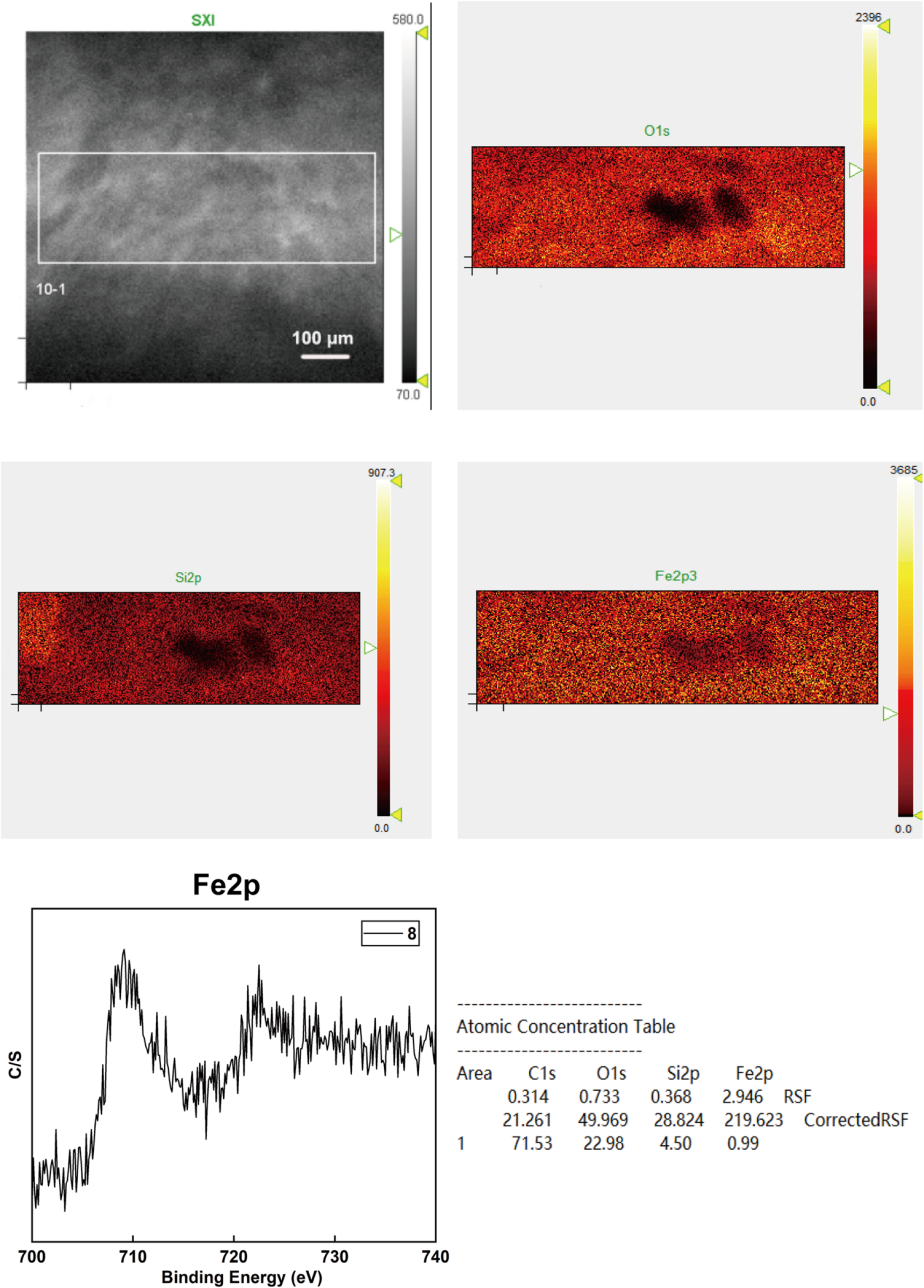
XPS mapping results of the PEO coating prepared in the Test 8.

Figure 6 shows XPS mapping results of the PEO coating fabricated in Test 8. XPS mapping is a more effective method than normal XPS spot scanning, as the scanning time is longer than that of normal XPS spot scanning, and each of the colored dots is a normal XPS spot scanning in the mapping. The XPS mapping result is the superposition of all the spot scannings. The atomic concentration is the average value of the scanning area. The XPS mapping results correspond with the results of GDOES. On the surface of the PEO coating, the content of Si is much higher than that of Fe. Furthermore, according to the fine spectrum of Fe2p, the PEO coating mainly contains Fe_3_O_4_ with a small amount of Fe_2_O_3,_ which also corresponds with the results of XRD^2^.

The general rule for the oxidation of Fe is as follows: the first product is FeO; then, the FeO can be oxidized to Fe_3_O_4_ very easily; at a higher temperature, the Fe_3_O_4_ will be oxidized to Fe_2_O_3_; at around 1535 °C, the Fe_2_O_3_ can be reduced to Fe_3_O_4_ again. Moreover, molten Fe_2_O_3_ and SiO_2_ in the plasma discharge channels can produce Fe_2_SiO_4_. At around 1600 °C, the Fe_2_SiO_4_ decomposes into FeO and SiO_2_^28,29^. Furthermore, the FeO has a loose structure, the Fe_3_O_4_ and Fe_2_O_3_ have a compact structure, but the Fe_3_O_4_ is more compact than Fe_2_O_3_. The descending order of thermal expansion coefficients is FeO, Fe_3_O_4_, Fe_2_O_3_, and Fe_2_SiO_4_^29^. The thermal expansion coefficient of SiO_2_ is around 0.5 × 10^–6^/°C. Q235 steel has a thermal expansion coefficient of about 12 × 10^–6^/°C, which is also higher than thermal expansion coefficients of Fe_3_O_4_, Fe_2_O_3_, and Fe_2_SiO_4_ at ambient temperature. Therefore, to achieve an excellent coating quality, it is necessary to form a compact and compatible transition layer between SiO_2_ and the steel substrate.

The compactness of the SiO_2_ coatings affects the breakdown voltage of PEO. Because Test 8 exhibits the highest breakdown voltage (170 V), the SiO_2_ coating produced in Test 8 is more compact than other coatings in phase A. Furthermore, the positive currents affect the PEO reaction temperatures. The temperature of PEO reactions determines the cooling speed of SiO_2_, which affects the crystallinity of SiO_2_ coatings. Under the higher frequency (2000 Hz) and a lower concentration of sodium silicate (19 g/L), Test 8 has the nearly lowest final positive current (8.5 A, see Table 3), and its reaction temperature is relatively low. Thus, the surface morphology of Test 8 is relatively smooth and has no obvious defects [see Fig. 3(8)].Besides, the PEO reaction temperatures affect the oxidation processes of Fe. Therefore, the PEO coating prepared in Test 8 has a compact and adaptive transition layer (mainly Fe_3_O_4_, as shown in Fig. 5), and an excellent coating quality as a whole.

Moreover, most of PEO coatings have a loose or thin transition layer, and cracks exist between the transition layer, the substrate, and the compact layer. For instance, the PEO coating prepared in Test 3 has a very thin transition layer [see Fig. 4(1)], which mainly contains FeO, and a little Fe_3_O_4_, Fe_2_O_3_ [see Fig. 5(3)]. The PEO coating prepared in Test 13 has a very loose transition layer [see Fig. 4(13)], which mainly contains Fe_2_O_3_, and a little Fe_3_O_4_ [see Fig. 5(13)]. The cracking is generated by the stress induced from different thermal expansion coefficients, and loose structure of FeO and Fe_2_O_3_. In conclusion, the specific phase composition is the key factor to affect the coating quality of PEO coatings.

### Potentiodynamic polarization measurements

**Figure 7:**
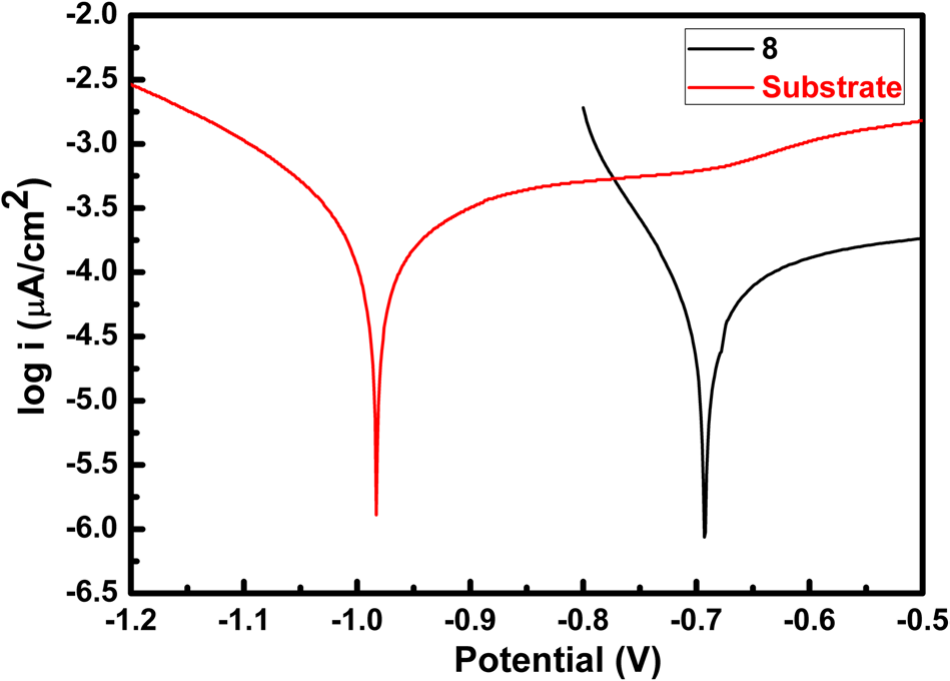
The potentiodynamic polarization curves of: Q235 steel substrate, and the PEO coating of Test 8.

Figure 7 displays the potentiodynamic polarization curves of the Q235 steel substrate and the PEO coating of Test 8.

The corrosion current density of the PEO coating of Test 8 is 52.6 μA/cm^2^, and its corrosion potential is − 0.693 V. The Q235 steel substrate has a corrosion current density of 244 μA/cm^2^, and a corrosion potential of − 0.983 V. After the PEO treatment, the corrosion potential of Q235 steel moves to a noble direction; meanwhile, the corrosion current density of PEO coating is 22% of bare Q235 sample. Therefore, the anti-corrosion property of Q235 steel is greatly improved by PEO surface treatments.

## Conclusion

In this work, a non-crystalline anticorrosive coating was prepared on Q235 low carbon steel by PEO. To promote the application of this technology in engineering practice and improve the coating quality, the coating-forming mechanism, elements and phase composition of the PEO coatings were analyzed deeply by an orthogonal experiment. The results indicated that:

1. The positive voltage is the main factor for preparing the anticorrosive PEO coatings.
2. The PEO process has similar characteristics with the traditional PEO process for Al, in terms of number and color changes of arcs, and surface crater-shaped morphology.
3. There are two layers between the coatings surface and the steel substrate, including SiO_2_ and a transition layer. The transition layer contains FeO, Fe_2_O_3_, Fe_3_O_4_, and Fe_2_SiO_4_; they can transform into each other during PEO processes. Furthermore, for the optimized PEO coating, the transition layer mainly contains Fe_3_O_4_.
4. The protocol of Test 8 of the orthogonal experiment produces an excellent PEO coating, which has the best coating quality, a corrosion current density of 52.6 μA/cm^2^, and higher corrosion potential of − 0.693 V.
